# Estimating the burden of United States workers exposed to infection or disease: A key factor in containing risk of COVID-19 infection

**DOI:** 10.1371/journal.pone.0232452

**Published:** 2020-04-28

**Authors:** Marissa G. Baker, Trevor K. Peckham, Noah S. Seixas

**Affiliations:** Department of Environmental and Occupational Health Sciences, University of Washington, Seattle, WA, United States of America; Massachusetts Department of Public Health, UNITED STATES

## Abstract

**Introduction:**

With the global spread of COVID-19, there is a compelling public health interest in quantifying who is at increased risk of contracting disease. Occupational characteristics, such as interfacing with the public and being in close quarters with other workers, not only put workers at high risk for disease, but also make them a nexus of disease transmission to the community. This can further be exacerbated through presenteeism, the term used to describe the act of coming to work despite being symptomatic for disease. Quantifying the number of workers who are frequently exposed to infection and disease in the workplace, and understanding which occupational groups they represent, can help to prompt public health risk response and management for COVID-19 in the workplace, and subsequent infectious disease outbreaks.

**Methods:**

To estimate the number of United States workers frequently exposed to infection and disease in the workplace, national employment data (by Standard Occupational Classification) maintained by the Bureau of Labor Statistics (BLS) was merged with a BLS O*NET survey measure reporting how frequently workers in each occupation are exposed to infection or disease at work. This allowed us to estimate the number of United States workers, across all occupations, exposed to disease or infection at work more than once a month.

**Results:**

Based on our analyses, approximately 10% (14.4 M) of United States workers are employed in occupations where exposure to disease or infection occurs at least once per week. Approximately 18.4% (26.7 M) of all United States workers are employed in occupations where exposure to disease or infection occurs at least once per month. While the majority of exposed workers are employed in healthcare sectors, other occupational sectors also have high proportions of exposed workers. These include protective service occupations (e.g. police officers, correctional officers, firefighters), office and administrative support occupations (e.g. couriers and messengers, patient service representatives), education occupations (e.g. preschool and daycare teachers), community and social services occupations (community health workers, social workers, counselors), and even construction and extraction occupations (e.g. plumbers, septic tank installers, elevator repair).

**Conclusions:**

The large number of persons employed in occupations with frequent exposure to infection and disease underscore the importance of all workplaces developing risk response plans for COVID-19. Given the proportion of the United States workforce exposed to disease or infection at work, this analysis also serves as an important reminder that the workplace is a key locus for public health interventions, which could protect both workers and the communities they serve.

## Introduction

As COVID-19 spreads globally, there is public health importance in characterizing the role of the workplace in disease transmission, given the variety of work tasks that could promote the spread of infectious disease (e.g., interfacing with customers, patients, and co-workers; preparing food), and the role of the workplace in spreading previous epidemics or pandemics [1,2].

It is known that those working in healthcare settings face increased exposure to agents causing infectious diseases such as SARS-CoV-2, but may also have better infectious disease protection plans and policies than other occupational settings, potentially limiting the transmission of disease to community members [3]. While important, these measures may be inadequate for the effective prevention of infection for such high risk occupations, especially when they are working with inadequate PPE stockpiles, and the hospitals are overwhelmed due to heavy patient loads [4]. Nearly 4% of confirmed COVID-19 cases in Wuhan, China (as of February 11, 2020) were in healthcare workers, indicating the workplace is a potential location of transmission even among workers who are trained to protect themselves from biological hazards [5].

However, other occupational groups which may have more sporadic exposure to infectious or disease-causing agents may not have the same level of planning, or even think that an infection disease control plan is warranted for their workplace. Of the first 25 COVID-19 cases confirmed in Singapore, 17 had probable relation to occupational exposure, including workers in retail stores and casinos, domestic workers, a tour guide, taxi and private hire car drivers, security guards, and workers at the same construction site, further exemplifying the role of the workplace in transmitting disease [6].

Understanding the burden of occupational exposure to infection and disease, including how many workers are potentially exposed and what occupations they work in, allows for upstream prevention measures, both at the workplace (e.g. developing appropriate infectious disease response plans, integrating infectious disease trainings into other workplace trainings, developing workplace policies that can support a workforce potentially exposed to SARS-CoV-2) and regulatory levels (e.g. increased access to paid sick leave, hazard pay for those exposed during a pandemic, etc.). These workplace and regulatory policies will be valuable in helping reduce the transmission of infectious disease from and within the workplace, and their importance may be realized with burden estimates

Previously, state-level employment data were utilized to estimate the number of workers exposed to a host of occupational exposures in United States Federal Region X (Washington, Oregon, Idaho, Alaska), spanning chemical, physical, ergonomic, and psychosocial hazards [7]. Here, utilizing the same data analysis methods as previously detailed in Doubleday et al., the number of workers across the United States exposed to disease or infection at work more than once a month is estimated. Despite some of the inherent limitations in using these existing data sources, we believe this analysis is valuable for informing risk assessments and prompting protective actions that occupational sectors and regulatory agencies can take during infectious disease outbreaks, such as COVID-19.

## Methods

Two sources of data were utilized for this analysis, and are detailed below.

United States employment data was obtained from the Bureau of Labor Statistics (BLS) Occupational Employment Statistics database [8]. The most current employment data at the time of analysis was from May 2018, and is organized by 2010 Standard Occupational Classification codes (2010 SOC). SOC codes are hierarchical, ranging from two-digits (Major Group Code) to six digits (Detailed Occupation Code), with the six-digit codes being the most detailed [9].

To estimate exposure to disease and infection in the workplace, we used data within the O*NET database. O*NET is a job characterization tool, generated from survey data, with rich information on tasks performed, skills needed, and job characteristics for different occupations, in order to inform job seekers or researchers [10]. As nearly 600 six-digit SOC occupations are updated each year, the entire O*NET database is completely refreshed every few years [11]. Between 2001 and 2011, nearly 160,000 employees from 125,000 workplaces had responded to O*NET questionnaires. O*NET uses a deliberate survey sampling scheme, to ensure representation of workers from across the United States, across organizations of different size, and from both government and private workers. For small SOCs where it may be hard to find respondents, and to complement data from job incumbents, O-NET also relies on occupational analysts and occupational experts to answer questionnaires. O*NET does not collect data from military occupations; thus, SOC codes beginning with 55 “Military Specific Occupations” are not included in O*NET data. Similarly, employment numbers for “Military Specific Occupations” is not reported in the BLS Occupational Employment Statistics Database. No other SOC codes are excluded from the O*NET database, but two SOC codes were not included in the measure utilized for this analysis, which were for the occupations of “Rock Splitters, Quarry” and “Timing Device Assemblers and Adjusters” employing 4,870 and 780 persons in the United States, respectively [12].

To characterize frequency of workplace exposure to infectious disease, we used the following O*NET question: “How often does your current job require you be exposed to diseases or infections?” Respondents, who take the survey online or on paper, could select from the following frequencies of exposure: Never; Once a year or more but not every month; Once a month or more but not every week; Once a week or more but not every day; Every day [13]. Respondents are given little context when completing the survey, with interpretation of the question up to the respondent. Within O*NET, these data are converted to a 0–100 score, representing weighted-average frequency of the metric for each SOC code. For this analysis, occupations were retained that had a score of 50–100, representing exposure to disease/infection more than once a month. SOC codes were merged with the national employment data to calculate the total number of workers employed in the occupations with exposure to disease/infection at more than once a month.

All data analysis was conducted using the statistical software package R version 3.6.3.

## Results

As of May 2018, there were a total of 144.7 million persons employed in the United States in employer-employee arrangements counted by BLS. Of these 144.7 million workers, an estimated 18.4% (26,669,810) were employed in occupations where exposure to disease or infection occurs more than once a month. As of May 2018, 10% (14,425,070) of the United States workforce was employed in occupations where exposure to disease or infection occurs at least once a week. Table 1 summarizes the number and proportion of workers exposed more than once a week and more than once a month by major occupational sectors (two-digit SOC). Both Healthcare Practitioner and Technical Occupations, and Healthcare Support Occupations have more than 90% of workers exposed more than once a month, and more than 75% of workers exposed more than once a week. Other notable major occupation groups with high proportion of exposure are Protective Service Occupations (52% exposed more than once a month, including police officers, firefighters, transportation security screeners), Personal Care and Service Occupations (52% exposed more than once a month, including childcare workers, nannies, personal care aides), and Community and Social Services Occupations (32.4% exposed more than once a month, including probation officers, community health workers, and social and human health assistants).

**Table 1:** Number and percent of workers exposed to infection or disease more than one time per month, and more than one time per week, by major (2-digit) Standard Occupational Classification code (SOC).

			Exposed > 1 time/month		Exposed > 1 time/week	
2-digit SOC		total in SOC	#	%	#	%
31	Healthcare Support	4,117,450	3,958,560	96.1%	3,160,890	76.8%
29	Healthcare Practitioners and Technical	8,646,730	7,911,430	91.5%	6,728,420	77.8%
33	Protective Services	3,437,410	1,789,490	52.1%	1,026,660	29.9%
39	Personal Care and Service	5,451,330	2,841,730	52.1%	29,810	0.5%
21	Community and Social Services	2,171,820	704,280	32.4%	168,190	7.7%
25	Education, Training, and Library	8,779,780	2,048,070	23.3%	--	--
37	Building and Grounds Cleaning and Maintenance	4,421,980	924,290	20.9%	--	--
43	Office and Administrative Support	21,828,990	3,532,530	16.2%	2,871,400	13.2%
19	Life, Physical, and Social Science	1,171,910	159,970	13.7%	20,030	1.7%
15	Computer and Mathematical	4,384,300	587,970	13.4%	--	--
53	Transportation and Material Moving	10,244,260	930,930	9.1%	118,770	1.2%
47	Construction and Extraction	5,962,640	491,990	8.3%	--	--
51	Production	9,115,530	371,480	4.1%	--	--
13	Business and Financial Operations	7,721,300	300,900	3.9%	300,900	3.9%
27	Arts, Design, Entertainment, Sports, and Media	1,951,170	57,140	2.9%	--	--
11	Management	7,616,650	59,050	0.8%	---	--
17	Architecture and Engineering	2,556,220	--	--	--	--
23	Legal	1,127,900	--	--	--	--
35	Food Preparation and Serving Related	13,374,620	--	--	--	--
41	Sales and Related	14,542,290	--	--	--	--
45	Farming, Fishing, and Forestry	480,130	--	--	--	--
49	Installation, Maintenance, and Repair	5,628,880	--	--	--	--
	**All SOCs**	**144,944,620**	**26,669,810**	**18.4%**	**14,425,070**	**10.0%**

The 16% of office and administrative support occupations with exposure to disease or infection more than once a month are patient representatives, couriers and messengers, and medical secretaries. The nearly 4% of workers exposed in business and financial operation occupations are compliance specialists, which includes environmental compliance specialists and coroners. The full O*NET dataset, ranking the frequency of exposure for each SOC is publicly accessible online [14], as is employment and wage data [8]. As these databases are periodically updated, they should be referenced for information on frequency of exposure for a specific occupation.

## Discussion

During an infectious disease outbreak, the workplace can play an important role in both spreading the disease [15,16] and helping to stop the spread of disease through workplace practices and policies [1,17]. Understanding the wide range of occupations that could be exposed to infection or disease due to work activities is important for planning risk management and communication to workers, in addition to prioritizing workplace response plans. This analysis estimates that the number of workers who face frequent exposure to an infection or disease at work; estimates of the number of workers who fall ill due to such exposures are not possible in this analysis. However, a primary goal of public health, especially in the face of a global pandemic, is to prevent the spread of disease. Therefore, understanding how many workers are frequently exposed, and what occupations they represent, is an important first step in being able to prompt and enact risk reduction strategies prior to disease transmission occurring, and illness manifesting. Thus, the results reported here have important public health implications.

Several limitations must be emphasized. Exposure to disease or infection in the workplace, and resultant transmission into the community, is dependent on many factors which were not able to be investigated in this analysis. This includes number of contacts that worker has with the public, workplace emphasis on and access to handwashing, number of interactions with bodily fluids, existing hygiene and cleaning practices in the workplace, availability of appropriate personal protective equipment (PPE) etc. While certainly this could vary between occupations, many of these factors would also vary within occupations, and none of these data were captured with the O*NET data. Presenteeism, reporting to work despite being symptomatic for disease, is common in the workplace, and is another contributor to the transmission of infectious disease, and potentially to the spread of epidemics or pandemics [2,18]. One analysis examined the role of workplace transmission in the 2009 H1N1 pandemic, estimating that about 8 million employees in the United States worked while infected, and that these workers may have caused the infection of as many as 7 million of their co-workers [19].

Access to paid leave, which could ameliorate the financial burden of staying home while sick, varies substantially by occupation, industry, employer, location, and worker sociodemographic profile (e.g., race/ethnicity) [20,21]. Workers without access to paid leave have higher rates of presenteeism, and are less likely to receive preventative health services such as getting flu shots [22]. Occupational sector also influences rates of presenteeism, with studies from various countries showing higher rates of presenteeism among workers in healthcare, public service, and educational sectors, as these essential services often do not have substitute workers available [23–25]. Indeed, a recent systematic review identified occupation type as one of the strongest predictors of presenteeism [2]. As many of these sectors are already exposed to disease due to work activities, it is important that disease response plans for these sectors include not only control methods to reduce exposures at work, but also contingency plans to ensure sick workers do not come back to work with disease. This could be accomplished through cross-training, providing extra paid sick leave during this time, ensuring flexible working conditions, and ensuring substitute workers are identified to fill in if essential workers fall ill.

Importantly, O*NET data are also subject to misclassification and undercounting. O*NET data were generated from self-reported subjective questionnaires and therefore are subject to bias and misclassification. Respondents may not realize they are exposed to infection or disease at work unless they are in a workplace where these hazards are communicated to them and protective equipment is provided (e.g., healthcare sectors) leading to potential differential misclassification across occupational groups. Workers could also be reporting expose to disease or infection that occurs while commuting to work (particularly by public transportation), leading to additional misclassification. Additionally, information from the O*NET database is applied at the occupation-level, and therefore does not account for within-job exposure variation [26]. Many workers are not included in the O*NET and BLS data sources, including independent contractors (which includes “gig economy” workers), domestic workers, self-employed, undocumented, and continent workers. These workers may be uniquely susceptible to exposure at work due to limited ability to take time off if they or a family member is ill [27]. In Sweden and Norway, higher rates of presenteeism (coming to work when sick) were found among low-income and immigrant workers [28]. This further emphasizes the importance of continuing to develop occupational surveillance systems that capture exposures and outcomes experienced by these undercounted groups, as well as ensure worker protections extend to protect these undercounted workers.

In conclusion, our analysis shows that a large proportion of the United States workforce, across a variety of occupational sectors, are exposed to disease or infection at work more than once a month. These are workers that public health should consider especially at risk for COVID-19, due to frequent exposure to disease and infectious agents. However, it should be noted that there are many other workers that could also be exposed to SARS-CoV-2, or encourage the spread of COVID-19, such as workers who are not given access to flexible working, workers who do not feel they can take sick time if they or a family member is sick, workers who do not have access to paid sick leave, or workers that perform essential services and do not have access to substitute workers. Work presented here underscores the importance of all workplaces developing sector-specific response plans to keep employees safe, halt the transmission of disease in the workplace, and ensure sick workers do not have to come to work. It also serves as a reminder that the workplace is an important locus for public health interventions, as many workers are frequently exposed to disease and infection at work, and their exposures can Increase disease incidence both in worker and community groups.
